# Laser Therapy as a Preventive Approach for Oral Mucositis in Cancer Patients Undergoing Chemotherapy: The Potential Role of Superoxide Dismutase 

**DOI:** 10.31557/APJCP.2021.22.10.3211

**Published:** 2021-10

**Authors:** Beatriz Coutens de Menezes, Marcela Marçal Thebit, Lucas André Silva Bonela, Karine Gadioli de Oliveira, Washington Luiz Gonçalves, Nazare Souza Bissoli, Carmem Luiza Sartorio, Sonia Alves Gouvea

**Affiliations:** 1 *Department of Physiological Science, Health Sciences Center, Federal University of Espirito Santo, Vitoria, Espirito Santo, Brazil. *; 2 *Biotechnology Post-Graduation Program, Health Sciences Center, Federal University of Espirito Santo, Vitoria, ES, Brazil. *; 3 *Residency Program in Health, University Santa Ursula, Rio de Janeiro, Brazil. *

**Keywords:** Oral mucositis, low-power laser therapy, solid tumors, chemotherapy, oxidative stress

## Abstract

**Purpose:**

Oral mucositis is a painful condition that occurs in patients who undergo chemotherapy. Due to the worsening of oral mucositis, the patient may progress to a worse clinical condition and interrupt antineoplastic treatment. There is little literature on low-power laser therapy in chemotherapy for other solid tumors. The purpose of this study was to investigate whether low-level laser therapy (LLLT) applied before chemotherapy could prevent oral mucositis in patients with solid tumors.

**Methods:**

Laser therapy was applied at a frequency of 630nm, with a dose of 2J / cm^2^, for the prevention of oral mucositis induced by chemotherapy specifically for non-hematological tumors. Epidemiological data, total neutrophils, general side effects, development of oral mucositis and degree, and the performance of low-power laser therapy to prevent oral mucositis were collected. The involvement of oxidative stress was evaluated by the enzyme superoxide dismutase (SOD) through blood samples, before and after chemotherapy treatments.

**Results:**

LLLT in the proposed protocol is efficient in reducing the development of oral mucositis (only at grade I/II) in patients under chemotherapy and able to reduce the severity of oral mucosal lesions, in patients who developed mucositis after the use of the laser for prevention. All individuals who underwent LLLT protocol did not show a significant reduction of SOD activity after the last chemotherapy cycle.

**Conclusions:**

The prophylactic laser therapy protocol proposed by the study, defined at a frequency of 630nm, a dose of 2J / cm2, demonstrated the ability to decrease the occurrence of oral mucositis in patients undergoing chemotherapy protocols to solid tumors. This effect could be related to preserved SOD activity, as it was observed that oral mucositis is related to leukopenia and reduced SOD activity and LLLT protocol prevented the decrease of SOD activity.

## Introduction

Chemotherapy is used in the therapy of control and remission of various solid tumors, such as those of the gastrointestinal tract, breast, and cervix. Its adverse effects are conditioned to the administration, the dose, and may involve hematologic, dermatologic and gastrointestinal tract alterations. Oral mucositis (OM) is considered one of the main side effects of chemotherapy for antineoplastic treatment. The prevalence of OM is variable, depending on the type of chemotherapy and individual response (Rubenstein et al., 2004; Curra et al., 2018), Keefe (2007) demonstrated that 5 to 15% of patients in chemotherapy could be affected by severe mucositis (grade III and IV). Depending on the grade, OM can lead to worsening of life quality during treatment, e.g. due to harder swallowing, hydric and alimentary intake, and capacity of communication. Moreover, the detrimental effects of OM may include also discontinuity of treatment (Parulekar et al., 1998; Bellm et al., 2000).

Several cytotoxic agents have been related to oral and gastrointestinal mucosal injury and some of them are especially associated with OM, e.g. methotrexate, fluorouracil, doxorubicin, cyclophosphamide, dactinomycin, bleomycin, and also the combined treatment with mitomycin, taxanes (paclitaxel and docetaxel), vincristine, and vinorelbine. These treatments can exacerbate toxicity in oral mucosa, leading to inflammation and ulceration (Moran, 2000; Curra et al., 2018). 

Many therapeutic approaches have been recommended to mitigate these adverse effects. Laser therapy has been considered a noninvasive technique that promotes pain relief and reduces the severity of oral mucositis in patients. There is evidence in the literature that low-level laser therapy in OM lesions results in a significant reduction of its severity, and promotes an important preventive effect on the appearance of lesions (Brugnera Júnior et al., 2003; Vladimirov Iu et al., 2004; Lubart et al., 2005; Stokman et al., 2006; Khouri et al., 2009).

The underlying mechanisms of photobiomodulation are not fully understood. However, in vitro and pre-clinical assays demonstrate that low-level laser therapy can mitigate apoptosis and improve cellular proliferation, migration (Nunez et al., 2012). Clinically, it may result in three relevant effects: analgesic, anti-inflammatory, and reparative, giving support to its therapeutic application. However, the type of cell, laser wavelength, and energy dose significantly influence these results (Nunez et al., 2012). 

Preventive usage of low-level laser therapy has been indicated for OM for patients who underwent radiotherapy in head and neck cancer (Antunes et al., 2013; Lalla et al., 2014; Zecha et al., 2016). Short term low-level laser therapy promotes the enhancement of reactive oxygen species (ROS), followed by induction of antioxidants, which counterbalance the redox equilibrium (Eichler et al., 2007). This antioxidant response may be a preventive response to oxidative stress, explaining the global antioxidant effect of low-level laser therapy (photobiomodulation) modulating the inflammatory cascade related to the induced aggression (Nunez et al., 2012). It has been demonstrated that antioxidant enzymes as superoxide dismutase (SOD) and catalase, which are inactivated in low pH and inflammation, are reactivated by laser light (Vladimirov Iu et al., 2004).

Despite laser treatment applicability in OM in patients with non-hematological tumors, there is a lack of information on its potential use as a preventive approach for adverse chemotherapy effects. In this context, the present work evaluated the preventive potential of low-level laser therapy (2 J/cm^2^) in patients with solid tumors who underwent chemotherapy. 

## Materials and Methods

The current retrospective study analyzed the data of 287 eligible patients with solid tumors. The study was approved by the Ethical Committee of the Federal University of Espírito Santo (CAAE: 2.186.172/2017), being conducted in accordance with the Helsinki Declaration. All participants signed the written informed consent form. A total of 287 patients with solid tumors, entre 2016 e 2018 in treatment in a reference oncology center (Centro Capixaba de Oncologia-CECON - Vitoria, ES, Brazil) were analyzed. The exclusion criteria were: patients who had already been treated for head and neck squamous cell carcinoma, or who had a recurrent malignant disease, or who were younger than 18 years of age. The demographic characteristics of the patients (gender, age, tobacco and alcohol consumption, tumor location, and tumor stage), were obtained from their medical records. The TNM stages of the tumors were determined according to the American Joint Committee on Cancer (AJCC) 7th edition staging system, using available clinical and pathologic data on tumor invasion, lymph nodes status, and metastasis. The body mass index (BMI) was accessed by measuring weight and height (kg/m2) and the systolic blood pressure (SBP) by using a validated digital blood pressure measuring device Omron HEM-705 CP (Omron Healthcare, INC. Illinois, USA).

*Treatments and laser therapy*

All patients enrolled underwent chemotherapy, in which AC-T represents the association of doxorrubicin and paclitaxel; TC is the association between docetaxel and cyclophosphamide; the associations of carboplatin and cisplatin are called PLATIN and the protocols using fluorouracil (FOLFOX, FOLFIRI, FEC) are called 5FU. The cancer patients were distributed into two groups: a group irradiated with laser emitting light in the red region (N = 204) and a control group – non- irradiated with laser (N = 83). 

The patients in the red laser group underwent a low-level laser irradiation application right before the starting of each cycle of chemotherapy. The DMC / LASER THERAPY XT (100mW) (Twin Laser – MM Optics S.A Ltda., São Carlos, SP, Brazil) apparatus was used, emitting a 630-nm wavelength, set at an output power of 30 mW, with beam area of 0.04 cm^2^ in the focal region. The laser dose was 2J/cm^2^, according Zecha (2016). The whole oral cavity was irradiated, including lip mucosa, soft palate, the floor of the mouth, buccal mucosa, tongue, and vestibule. Each point in the irradiated area was at a distance of about 1 cm from the other irradiation points.

*Biochemical Analysis*

*Blood samples*

From all patients enrolled, 35 individuals (which were starting chemotherapy at the beginning of the study) had blood samples collected. A blood sample (10 mL) was collected from each patient in two different time points - before starting chemotherapy and in the last cycle of chemotherapy. All samples were centrifuged (4°C, 2,000g, 15 min) and serum samples were aliquoted and stored at -80°C for subsequent analysis. An aliquot of blood was designed for laboratory leucocyte count.

*Serum biochemical assays*

Superoxide Dismutase (SOD) activity was assessed using a kit (Invitrogen, ThermoFisher Scientific), following the manufacturer’s instructions. SOD radicals generated by xanthine oxidase and hypoxanthine were detected with tetrazolium salt. One unit of SOD was considered as the amount of enzyme that promotes a 50% dismutation of superoxide radical. Substrate (50 µL) was added to 10 µL of samples (diluted 5x) and standards (in duplicates) in a 96-well plate. Xanthine oxidase (25 µL) was added to initiate the reaction. The absorbance was read at 450 nm after 20 min incubation at room temperature on a shaker using a plate reader. Quantification was performed by comparison with standard SOD concentrations, being expressed in U/ml. 

*Statistical analysis*

Data are reported as means±SD. Kruskal-Wallis test was used to perform comparisons of averages between leucocyte counting and mucositis. Mann-Whitney test was used to compare SOD activity, oral mucositis, and laser therapy. Qualitative variables were analyzed through the chi-square test and Fisher’s exact test (for samples in which n<5). Data were analyzed with the Statistical Package for the Social Sciences (SPSS®, version 20). A 95% confidence interval and a significance level of 5% (p< 0.05) were considered.

## Results

The descriptive features of the patients were grouped regarding age, gender, hemodynamics, body composition and anthropometry, and blood pressure. The majority of selected individuals were women (79.8%, N=229 vs. male 20.2%, N=58), and the mean age was 56 years (Range 29-89). The consumption of tobacco and alcohol was observed in a few individuals of the sample. However, the majority were non-smokers 94.4% (N = 271) and non-alcoholics 87.5% (N = 251). Regarding comorbidities, only 31% of all patients presented Hypertension and 16.7% Diabetes. 

As a complementary evaluation general parameters as arterial blood pressure, and body mass index (BMI) were measured before and after chemotherapy. No alterations were observed in these parameters at the end of the treatment. The mean initial systolic/diastolic pressure (123/78 mm Hg) was similar to the end of treatment (119/76 mmHg). The initial BMI was 25.89 and the end of 25.75. 

The majority of patients enrolled presented breast (62.2%) or gastric (25.4%) cancer. All patients underwent chemotherapy, and the largest part was treated with AC-T and 5FU. The TMN Classification of Malignant Tumors demonstrated that more than 50% of tumors, at diagnosis time, were in the initial stage (I and II), independent of their locations (Table 1).

Adverse effects during chemotherapy were evaluated regarding the frequency of occurrence, and protocol of treatment (Table 2). From 287 patients, 277 (96.5%) showed at least one adverse effect during chemotherapy cycles. Considering all patients analyzed, nausea was the most frequent symptom (57.4%) and oral mucositis showed a high incidence (30.6%).

No differences were observed in the manifestation of OM when analyzed different protocols of chemotherapy (AC-T, TC, 5FU and PLATIN; p=0,384). However, without laser therapy 69.9% of the individuals evaluated developed OM. 

To prevention and treatment of OM low-level laser therapy was used (frequency of 630nm –dose 2J/cm^2^). The results of 204 patients analyses who underwent preventive LLLT, demonstrated that this protocol can reduce occurrence and gravity of OM (Table 3). Patients who underwent laser therapy presented OM only at grade I and II, with the majority at grade I. However, patients without laser therapy presented OM grade I to III, and most frequently the grade II (Table 3). Moreover, considering individual protocols of treatment and OM occurrence, laser therapy showed a significant reduction difference in the manifestation of oral mucositis as compared to patients who did not undergo laser therapy.

**Figure 1:**
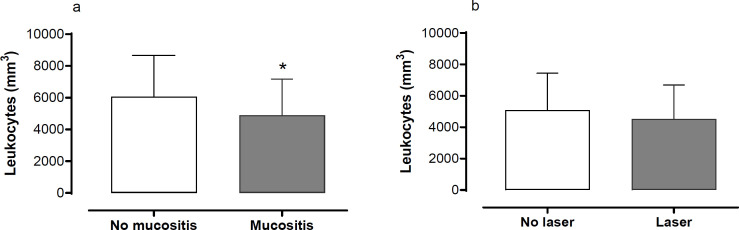
Leucocyte Count and Oral Mucositis. (a) Total leucocyte count and development of oral mucositis, (b) Total leucocyte count in patients with oral mucositis, with or without laser therapy. Mann-Whitney test; *p<0.0001. Data are mean ± SD

To evaluate the potential causes related to this effect of laser therapy leucocyte counting analysis was performed in patients with or without OM (Figure 1). Leucocyte counting is significantly lower (p<0.05) in patients presenting oral mucositis (Figure 1a). On the other side, laser therapy did not change leucocyte quantity in patients presenting OM (p=0.147, Figure 1b).

**Figure 2:**
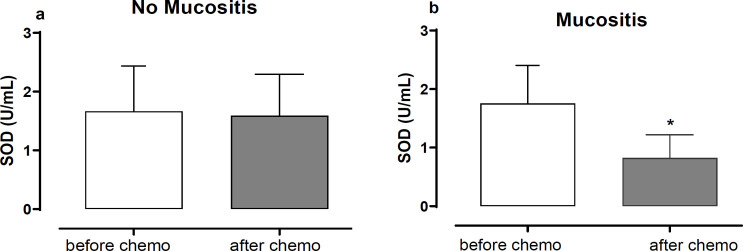
Superoxide Dysmutase (SOD) Activity in Serum of patients (a) without mucositis or (b) with mucositis before the first and after the last cycle of chemotherapy. Mann-Whitney test; *p<0.05. Data are mean ± SD

Moreover, from all patients included in this study, 35 individuals underwent blood withdrawn to evaluate superoxide dismutase (SOD) activity – before the first and after the last cycle of chemotherapy. No differences were observed in serum SOD activity in patients without mucositis (1.664±0.77/ 1.589±0.70 U/mL Before/After Chemo; p=0,693; Figure 2a). On the other side, in patients with mucositis SOD activity was reduced (p<0.05) at the end of treatment (1.753±0.65/ 0.824±0.0.39 U/mL, Before/After Chemo; Figure 2b). 

**Figure 3:**
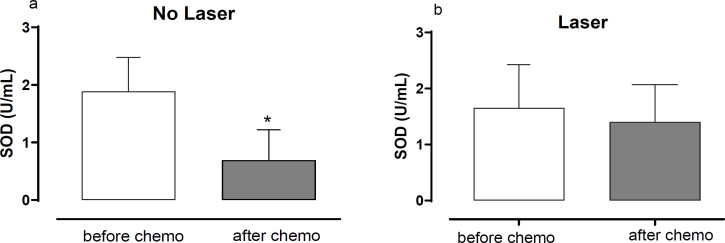
Superoxide Dismutase (SOD) Activity before the First and after the Last Cycle of Chemoterapy in Serum of Patients (a) whithout laser therapy or (b) who underwent laser therapy. Mann-Whitney test; *p<0.05. Data are mean ± SD

In addition, the level of SOD activity in the serum of patients who underwent or not laser therapy – before the first and after the last cycle of chemotherapy was compared (Figure 3). In patients not submitted to laser therapy, there was a significant reduction (*p<0,05) of SOD activity (1.89±0.66/ 0.691±0.53 U/mL Before/After Chemo; Figure 3a) when comparing SOD levels before the first and after the last cycle of chemotherapy. Interestingly, laser therapy was able to prevent the reduction of SOD activity at the observed time points (1.652±0.77/ 1.404±0.66 U/mL Before/After Chemo; Figure 3b).

**Table 1:** Location, Stage and Regimen of Chemotherapy Treatment

	N (%)
Solid tumors	
Breast	190 (66.2)
Gastrointestinal tract	73 (25.4)
Pancreas	12 (4.1)
Uterus/ovarian	4 (1.4)
Lung	2 (0.6)
Prostate	2 (0.6)
Larynx	2 (0.6)
Liver	1 (0.3)
Vesicle	1 (0.3)
Conditioning regimens	
AC-T	139 (48.4)
5 FU	95 (33.1)
TC	43 (15)
PLATIN	10 (3.5)
T	
1	68 (26)
2	93 (35.5)
3	74 (28.2)
4	27 (10.3)
N	
0	121 (48)
1	101 (40.1)
2	26 (10.3)
3	4 (1.6)
M	
0	206 (82.1)
1	45 (17.9)
Stage of disease	
Stage I	61 (21.6)
Stage II	99 (35.2)
Stage III	68(24.1)
Stage IV	54 (19.1)

ACT, doxorrubicin+cyclophosphamide+paclitaxel; 5FU, fluorouracil; TC, docetaxel+cyclophosphamide; PLATIN, carboplatin and cisplatin

**Table 2:** Adverse Effects of Chemotherapy Protocols

	Diarrhoea N (%)	Nausea N (%)	Vomiting N (%)	Mucositis N (%)
AC-T	17 (12.2)	97 (69.7)	25 (18)	45 (32.3)
5 FU	39 (41)	39 (41)	20 (21)	32 (33.6)
TC	14 (32.5)	23 (53.5)	05 (11.6)	09 (20.9)
PLATIN	02 (20)	06 (60)	01 (10)	02 (20)

(doxorrubicin+cyclophosphamide+paclitaxel), 5FU (fluorouracil), TC (docetaxel+cyclophosphamide), PLATIN (carboplatin and cisplatin).

**Table 3:** Comparison between Preventive Laser Therapy and the Development of Oral Mucositis in Different Protocols. In sequence, the grades (I, II, III and IV) of the subjects (N = 88) with mucositis who did not use laser therapy were evaluated with those who did

	No Laser N (%)	Laser N (%)	P
AC-T			
No Mucositis	11 (27.5)	83 (83.9)	p<0.0001
Mucositis	29 (72.5)	16 (16.1)	
TC			
No Mucositis	02 (25)	32 (91.5)	p=0.0003*
Mucositis	06 (75)	03 (8.5)	
5 FU			
No Mucositis	11 (33.3)	52 (83.9)	
Mucositis	22 (66.7)	10 (16.1)	p<0.0001
Grade Mucositis			
I	19 (32.75)	22 (73.3)	
II	30 (51.75)	08 (26.7)	p<0.001
III	09 (15.5)	0	
IV	0	0	

AC-T, doxorrubicin+cyclophosphamide+paclitaxel; 5FU, fluorouracil; TC, docetaxel+cyclophosphamide

## Discussion

This work demonstrates the potential preventive effect of laser therapy for oral mucositis in patients with solid malignant tumors who underwent chemotherapy. This protection could be related to the maintenance of SOD activity in patients receiving laser therapy.

In the present study, the majority of patients were women, and the most common tumor was breast cancer, followed by gastrointestinal tumors. Previous reports indicate that in women at the same age of the patients included in the present work, breast cancer is the most common tumor (INCA, 2019).

Staging at diagnosis time is variable, and it directly influences the treatment choice and prognosis. In this study, the majority of the patients presented tumors at stage II. Associated to stage I, these are considered initial states of cancer. However, previous studies demonstrated that more advanced stages (III and IV) are more commonly found in Brazil at diagnosis time (INCA, 2020). This discrepancy probably is related to the sample, as our patients were from a private clinic, having a different profile regarding the feasibility of early diagnosis.

Oral mucositis has been described as one of the adverse effects related to chemotherapy, and the search for preventive and therapeutic approaches is relevant to manage this clinical feature. In the present study, the chemotherapic compounds were mostly AC-T (48.4%) and 5FU (33.1%). From this sample, 32.3% of which treated with AC-T and 33.6% which received 5FU presented mucositis. Al Ibraheemi (2016) have demonstrated 89.3% of mucositis in patients treated with AC-T, independent of the gravity of tumor and without preventive approaches. Epstein (1999) also demonstrated the association of chemotherapy and oral mucositis. From all adverse symptoms associated with chemotherapy in the current study, mucositis represents 30.6% of incidence, independent of treatment choice.

However, in patients who underwent laser therapy, mucositis incidence was lower (14.7%) as compared to patients not submitted to this treatment (69.9%), and this reduction was observed in all chemotherapic protocols analyzed. In the current study, the application of low-level laser therapy in the red spectrum (630nm - 2J / cm^2^), in all cycles of chemotherapy, significantly reduced the severity of oral mucositis. Moreover, in patients who underwent laser therapy and still showed mucositis, it was less severe - grade I and II - with no manifestation of mucositis grade III or IV.

Laser therapy has been described as a treatment in patients with malignant neoplasm of the head and neck to mitigate the effects of mucositis due to chemoradiation therapy (Zecha et al., 2016). The use of photobiomodulation in hematopoietic stem cell transplantation patients is also documented, related to high-dose chemotherapy in hematological malignancies, being effective in reducing the severity of chemotherapy-induced oral mucositis (Eduardo Fde et al., 2015; Silva et al., 2015; Bezinelli et al., 2016). Despite the benefits of inducing photobiomodulation by laser therapy in patients with solid tumors undergoing chemotherapy, there is no consensus regarding laser doses and frequencies to be used, which can vary from 7 to 10 days of application and in doses that vary from 2 to 10 J / cm^2^ (Anschau et al., 2019; Daugėlaitė et al., 2019).

NetoWestphalen (2018) described prophylactic laser therapy in chemotherapy for solid tumors with laser application frequency and doses different from those used in the present study. These authors evaluated the use of low-level laser therapy to reduce the severity of oral mucositis in individuals with breast cancer and receiving chemotherapy [fluorouracil (5-FU) + adriamycin (doxorubicin) + cyclophosphamide], using 660nm laser - 4J / cm^2^ or 808nm - 4J / cm^2^, during 7 consecutive days. The severity of mucositis in the laser group was lower than in the control group, which used only 0,12% chlorhexidine, but no statistical difference was observed between the two wavelengths appointed. These results demonstrate that low-intensity laser radiation can be useful in the treatment of oral mucositis. Our results demonstrate that laser therapy, even in lower doses (630nm - 2J/cm^2^), has shown preventive effects reducing the incidence and gravity of oral mucositis related to chemotherapy. 

On the other hand, Rozza-de-Menezes (2018) demonstrated that in patients under treatment with fluorouracil and doxorubicin for solid tumors, both the use of low-power laser therapy (650nm, 4J / cm^2^) or improvement of oral care showed positive results to prevent oral mucositis and should be further investigated in similar and larger samples.

The presence of mucositis has been associated with leukopenia, due to the reduced capacity for an inflammatory response to the cytotoxic effects of chemotherapy on the oral mucosa, which may alter the final response of tissue repair (Suresh et al., 2010; Patussi et al., 2014; Al Ibraheemi and Shamoun, 2016). According to Suresh (2010), leukocyte levels below 3,000 / mm^3^ increase the risk of oral mucositis. In the present study, we corroborate this observation where the reduction in the number of total leukocytes occurs in parallel with the higher incidence of oral mucositis, and the use of low-level laser therapy did not change this parameter. This fact must be related to the impairment of the inflammatory response, mediated by immune cells, impairing the inflammatory, proliferative (mediated by fibroblasts, associated with collagen production and angiogenesis), and remodeling (reorganization and maturation of the tissue) phases of healing (Martin and Leibovich, 2005). Given the above, the lack of effect of low-level laser therapy photobiomodulation on leukocytes could point to other basal mechanisms involved in this response.

The relationship between the development of mucositis and oxidative stress has been described, by the formation of reactive oxygen species (ROS) that maintain mechanisms of cellular damage, caused initially by the cytotoxicity of chemotherapy protocols (Scully et al., 2006). In the present study, no change in SOD activity was observed before the start of treatment and in the last chemotherapy cycle in those patients who did not present mucositis. The decrease in SOD activity with the use of cytotoxic chemotherapeutic agents has already been described, leading to an increase in oxidative stress and leading to an increase in adverse effects (Sakanyan, 2018). In this context, measures that can preserve or increase the redox balance could prevent the adverse effects of chemotherapy (Borutaite et al., 2000; Bezinelli et al., 2016; Dos Santos et al., 2017; Anderson et al., 2018).

In the present study, patients who developed oral mucositis showed a reduction in serum SOD activity in the last chemotherapy cycle when compared to the beginning of treatment. However, when only patients with mucositis were observed, prophylactic low-level laser therapy was able to prevent the reduction of SOD. The possibility of low-level laser therapy being related to maintaining or increasing SOD activity was raised by Vladimirov Iu (2004) in an analysis of the mechanisms of action of low-level laser therapy in different tissues and cells. This fact was also reported in other pathological conditions such as experimental rheumatoid arthritis, where there is an increase in the production of ROS, and laser therapy was associated with an increase in antioxidant defenses, including SOD. The current study brings new information regarding the effect of photobiomodulation by low-level laser therapy and SOD activity, acting as prophylactic approach for oral mucositis related to chemotherapy. 

In conclusion, the prophylactic laser therapy protocol proposed by the study, defined at a frequency of 630nm and a dose of 2J / cm^2^, demonstrated the ability to decrease the occurrence of oral mucositis in patients undergoing chemotherapy protocols with high potential to induce tissue damage, in patients with non-hematological (solid) tumors. It was observed that oral mucositis is related to leukopenia and reduced SOD activity. Low-level laser therapy prevented the reduction of SOD activity, without influencing leukocyte levels. Photobiomodulation by applying low-power laser could be a non-invasive approach in the prevention of oxidative stress, mitigating the damaging effects of chemotherapy such as oral mucositis. Its clinical applicability has been demonstrated as an important therapeutic tool and its novel preventive potential is demonstrated by this study.

## Author Contribution Statement

BCM, MMT: equally contributed to conception, design, analysis and interpretation of data. KGO, LASB, and WLG: contributed to acquisition of data and literature research. CLS, NSB and SAG: provided resources and final approval of the manuscript. KGO, CLS and SAG: contributed to the discussion and manuscript drafting. All authors have read and approved the final version of the manuscript.
